# Two-dimensional spreading properties and sealing characteristics of fluorocarbon surfactants on several typical hydrocarbon fuels

**DOI:** 10.1038/s41598-020-80932-8

**Published:** 2021-01-13

**Authors:** Xuhong Jia, Yuzhen Luo, Rui Huang, Xinhua Zhu, Yuqiang Zhang, Quanyi Liu

**Affiliations:** College of Civil Aviation Safety Engineering, Civil Aviation Flight University of China, Guanghan, China

**Keywords:** Chemistry, Engineering

## Abstract

A new method for studying the two-dimensional spreading properties and sealing characteristics of surfactant solution on oil surface was provided. The actual spreading situation of the C4-Br/oil systems in axisymmetric geometry was observed directly using HD camera for the first time and the results showed that the aqueous film expanded outwards in a circle with the guiding device as the center. Meanwhile, the relation between spreading radius and time was investigated and evaluated using the model for surface-tension-viscous regime. The root-mean-square deviation (*RMSD*) values obtained from the correlation for all of the systems we studied below 1.64, indicating a good agreement between the experimental and theoretical values. The results of sealing experiments showed that the aqueous film could absolutely seal the oil surface for 27–65 s and the sealing effect would be lost after 216–742 s for different systems. The stronger the volatility was, the shorter the sealing time was. Additionally, the volume percentage of oil vapor with film was always lower than that without film even when the evaporation was saturated. These findings were of great significance to guide the preparation of efficient AFFF.

## Introduction

As one of the most effective extinguishing agents for oil fires, aqueous film- forming foam (AFFF) has double extinguishing functions of aqueous film and foam^1–7^. The aqueous film can prevent evaporation of oil and isolate oxygen, and the foam can shield the thermal radiation of the flame and further reduce the fuel evaporation. Aqueous layer plays an important role in the fire fighting process. However, the importance of aqueous film has not been paid enough attention since the appearance of AFFF. The standards of AFFF, such as international standard^8^, EU standard^9^, US standard^10^ and Chinese standard^11^ have not specified its spreading property. In particular, the study on the spreading dynamics of aqueous film on the oil surface has been rare for decades and only a few studies have reported the relationship between the spreading performance and the fire extinguishing effect of aqueous film.

The study on the spreading kinetics of aqueous film formed by surfactant solution on oil surface is mainly seen 20 years ago or even earlier. In 1926, Landt and Volmer^12^ proposed that the spreading distance and spreading time of a liquid on another incompatible liquid surface obeyed power law. Decades later, Fay^13,14^ demonstrated that the relation between spreading length and spreading time of the oil film on water could be described by the following equation,

1 $$L = k\left( {\frac{{s^{2} }}{{\rho^{2} \upsilon }}} \right)^{\frac{1}{4}} t^{\frac{3}{4}}$$

where *L* was the length of spread, cm; *k* was a constant characteristic of the given system; *S* was the spreading coefficient, mN/m; *ν* was the kinematic viscosity of fuel, cm^2^/s; *ρ* was the density of fuel, g/cm^3^; *t* was the spreading time, s. Joos and Hunsel^15^ found that the spreading kinetics of fluorocarbon surfactant (FC-129) solution on the surface of carbon tetrachloride obeyed Eq. (1), but for the mixture solution of perfluoroammonium caprylate and cetyltriammonium bromide surfactant spreading on benzene, the spreading rate is proportional to 0.575 power of time instead of 0.75. Jho^5^ studied the spread of a commercial AFFF agent (FC-206A) on the mixtures of *n*-heptane and *n*-hexane surface, and found that the average spread rate the average areal spreading rate was directly proportional to the equilibrium spreading coefficient. Also, it was pointed out that the spreading rate was determined by the dynamic spreading coefficient and the concentration of the solution. Dussaud and Troian^16^ suggested that the spreading rate of liquid films on a deep water layer for hydrocarbons obeyed a power law in time, where the power exponent for nonvolatile film was 0.75 but for volatile immiscible film closed to 0.5.

Some studies have also reported the repression of aqueous film on the volatilization of hydrocarbon fuels. Moran et al*.*^2^ found that fluorochemical surfactant films with sufficient thickness could reduce the rate evaporation of cyclohexan and JP-4 jet fuel by 90 to 98%. Nicolson and Artman's research^17^ showed that the An sul AFFF solution spread on cyclohexane could achieve a 98% seal and maintained it for at least 1.5 min. Ranjbar and Shahraki^18^ in their study pointed out that the insoluble films of aqueous surfactants could, but soluble ones could not reduce the evaporation rate of the fuel. Schaefer et al.^19^ explored the relationship between the formation of aqueous film and the fire extinguishing performance of AFFF. They found that the spreading property of thin films of fluorosurfacants solution was not as important for fire suppression as the improved sealability of flammable vapours. Williams et al.^20^ carried out a large-scale (28 ft^2^) fire-extinguishing experiments according to the U.S. DoD MilSpec procedure, and separated the effect of film formation on fire extinguishment performance from other properties of AFFF. The result showed that the fire extinguishing times of film forming foam extinguishing agents would be 5–12 s shorter than that could not form film and the film formation contributed to good AFFF fire extinguishment performance by 20%.

In views of their excellent properties, such as high thermal stability, excellent surface activity and great advantages in fire extinguishing performance, the fluorocarbon surfactants are used as a key ingredient in AFFFs^21–23^. For a long time, AFFFs based on 8-carbon chain fluorocarbon surfactants (C8) had dominated the foam extinguishing agent market. However, C8 had been replaced gradually by the short carbon chain ones due to its threat to human health and environment^24–26^. Considering its excellent film-forming and fire-extinguishing properties, and its toxicity and bioaccumulation can be ignored, C_4_F_9_SO_2_NH(CH_2_)_2_ N(CH_3_)_2_CH_2_Br-CH_2_OH (C4-Br), which has a broad application prospect in AFFF, is selected as the surfactant to study its spreading performance^27^. It's worth noting that the spreading kinetics of fluorocarbon surfactants with different carbon chain lengths on several typical liquid fuels in one-dimensional direction had been studied in our previous work^28^. However, the aqueous film of AFFF spread in two dimensions in the actual fire extinguishing process, and the standards of foam fire extinguishing agents developed by various countries and organizations were also based on pool fire^8–11^. Therefore, the spreading performance of fluorocarbon surfactant solutions on the surface of hydrocarbon fuels in two-dimensional direction was studied in this work. In addition, the sealing performance of aqueous films with different thickness for hydrocarbon fuels was also studied.

## Experimental section

### Materials and instruments

The fluorocarbon surfactant C4-Br was synthesized using the method reported in our another work^27^. Sodium hexanesulfonate and methylene blue were provided by Shanghai Macklin Biochemical Co., Ltd. Cyclohexane and *n*-heptane produced by Chengdu Cologne Chemical co., Ltd. Diesel oil 0# and aviation kerosene were provided by China National Aviation Fuel Grope. Solvent naphtha purchased from Hubei Xin Bonus Chemical Co., Ltd. All reagents were used without further purification. All aqueous solutions were prepared using deionized water.

Tensiometer (model A601) was produced by USA Kino Industry Co., Ltd. The oil pan (fire tray) was made of stainless steel by ourselves according to international standards^8^. High-definition camera (model D810) made by Nikon Corporation. Flammable gas detector (model MS400) was produced by Beijing Sipute Technology Co., Ltd. Kinematic viscosimeter (model SYD-265C) was provided by Shanghai Changji Geological Instrument Co., Ltd. Capillary-stoppered pyknometer (model 25 mL) was produced by Fuzhou Beibo Experimental Instrument Co., Ltd. Thermogravimetric analyzer (Perkin-Elmer TGA 4000) was made by PerkinElmer Co., Ltd..

### Experimental procedure

#### Spreading experiment

**Figure 1:**
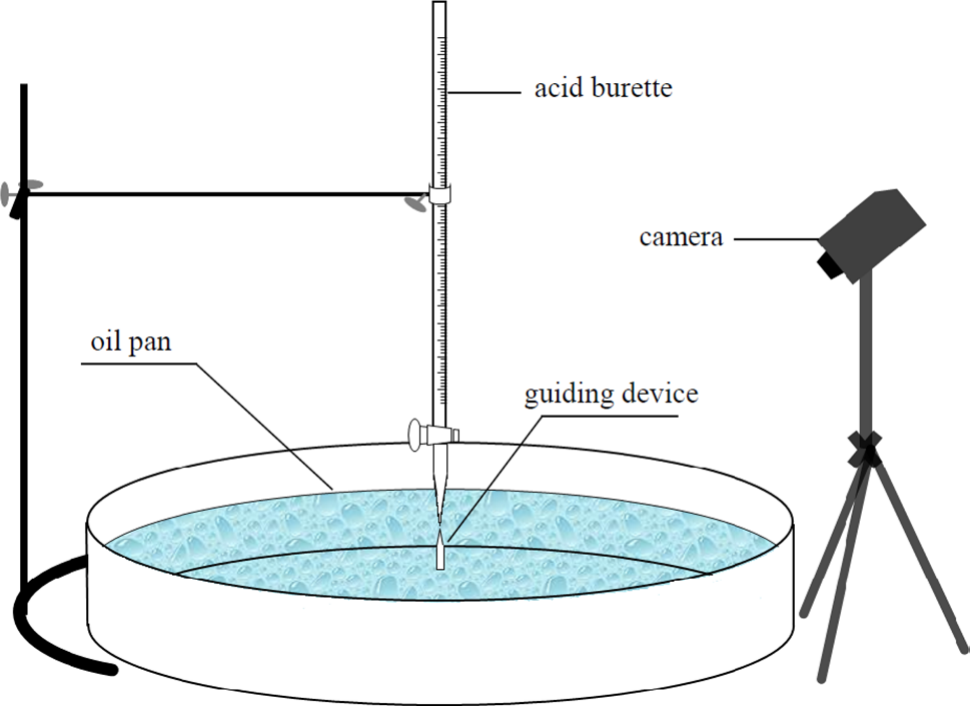
Schematic diagram of spreading experimental device.

As shown in Fig. 1, the experimental device for obtaining two-dimensional spreading dynamics data was self-made. The round oil pan was made of stainless steel with dimensions of internal diameter at rim 565 mm and height of vertical wall 150 mm according to the Annex H in ISO 7203–3:2011(E)^8^. A needle like guiding device was arranged at the center of the oil pan to determine the center position and reduce the interference of gravity on the flow of liquid when adding the solution. Just above the center was an acid burette with a measuring range of 10 ml. Add surfactant solution to acid burette and carefully adjust its concave level to zero scale for standby. There were two layers of liquid in the oil pan. At the beginning of the experiment, the upper layer of the pan was filled with 2.5 L hydrocarbon oil (about 1 cm thick) and the lower one was filled with 2.5 L water (about 1 cm thick) dyed with Methylene Blue to observe the spread process of aqueous film more clearly. Then, fixed the burette filled with solution vertically above the center of the circle, and make its outlet almost contact with the upper end of the guiding device. Rotated the burette piston to a certain open angle to ensure the liquid had a same flow rate in each experiment. Finally, the spread time and location of the aqueous film could be recorded by high-definition camera. All of the solutions and fuels used in the experiments were kept at 25 °C more than 4 h in incubator and the temperature of the experimental ambient was maintained constant at about 25 °C by air conditioner. The test was repeated at least three times and the average measurements were used for statistical analysis of data.

#### Sealing experiment

**Figure 2:**
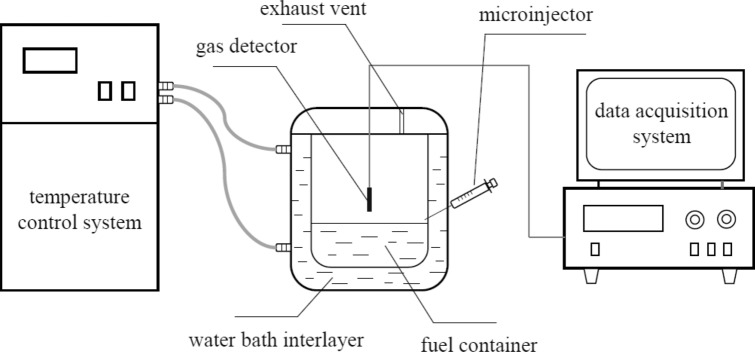
Schematic diagram of sealing experimental device.

As shown in Fig. 2, the sealing experimental device made of glass was used to obtain the volume percentage of fuel vapor. The cylindrical fuel container with water bath interlayer was 90 mm in inner diameter and 100 mm in height. The small hole with a plug on the side of the container was used to fill surfactant solution by a microinjector. Flammable gas detector was accessed through the cover of the container with a 5 mm diameter vent to atmosphere. Firstly, inhaled the surfactant solution with a certain concentration into the microinjector and adjust it to zero scale for standby. Next, started the temperature control system adjusted to 25 °C in advance and inserted microinjector into side hole for use. After 10 min, added 200 ml of fuel preheated to 25 °C into the container using a measuring cylinder. Immediately, started the flammable gas analyzer and injected surfactant solution. Data acquisition system could collect the data of volume percentage of the flammable gas every second. The average value of at least three experiments was used for data analysis.

### Test methods

#### Test of surface tension and interfacial tension

The surface tension and interfacial tension were measured using Lecomte du Noüy method^29^ at (25.0 ± 0.1)°C in a thermostat. The average readings of three measurements were used for the graphs.

#### Test of density and kinematic viscosity

The density of the hydrocarbon oils used in this work was measured according to the international standard ISO 3838–2004^30^ and the kinematic viscosity was tested follow with the Chinese national standard GB/T 265–88^31^, respectively. The test was conducted at least three times, and the average value was used for data analysis.

#### Test of aqueous film thickness

The thickness of aqueous film formed by surfactant solution was investigated using a cylindrical glass dish with diameter of 180 mm and height of 25 mm. Took 1000 µL of pre-prepared solution with concentration of 10.0 mmol/L using a microinjector, dropping in the dish center very slow and uniform, and observed the spread of aqueous film on the oil surface carefully. The dosage of the solution when the film just covered the oil surface was recorded as the minimum spreading amount (*V*_min_). Continue to add solution until the first solution drop sank from the oil surface and recorded the dosage as the maximum spreading amount (*V*_max_). The minimum and maximum film thicknesses (*σ*_min_ and *σ*_max_) could be calculated according to the *V*_min_, *V*_max_ and the area of the dish respectively. The experiment was repeated at least three times at an ambient temperature of 25 °C.

#### Test of the thermal stability for the C4-Br

**Figure 3:**
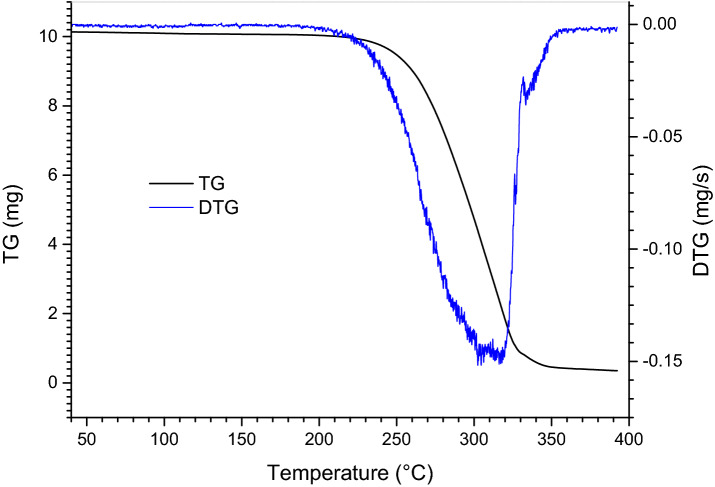
TG and DTG curves of surfactant C4-Br.

Considering that surfactant would decompose and lose their performance at high temperatures and the aqueous film spreads on the hot oil surface in the actual fire extinguishing process, the thermal stability of C4-Br was tested. TG analysis of C4-Br was carried out with Pyris thermogravimetric analyzer. Approximately 10 mg of the sample was heated from 40 to 800 °C at a heating rate of 10 °C/min under air atmosphere. As shown in Fig. 3, the sample maintained its weight below 230 °C, showing a good thermal stability.

## Results and discussion

### Two-dimensional spreading properties

#### Relationship between spreading radius and time

Spreading properties of surfactant solution on oil surface was affected by many factors, such as surface tension of substrate, concentration and surface tension of surfactant solution, interfacial tension between oil and water, kinematic viscosity and density of substrate, etc. Since the effect of the tail chain length of surfactant on its spreading performance had been clarified^22^ and the short fluorocarbon chain surfactants had a wide range of application prospects due to their environmental advantages^32–35^, the C4-Br/CH_3_(CH_2_)_5_NaO_3_S equimolar system was chosen as the aqueous solution in this work. The concentration of the surfactant system was set as 10.0 mmol/L for that, on the one hand, the surfactant concentration in industrial application was most slightly higher than its CMC value (7.25 mmol/L for C4-Br/CH_3_(CH_2_)_5_NaO_3_S system)^27^, and on the other hand, it was convenient to compare with the results of the one-dimensional spreading performance in our previous study.

**Figure 4:**
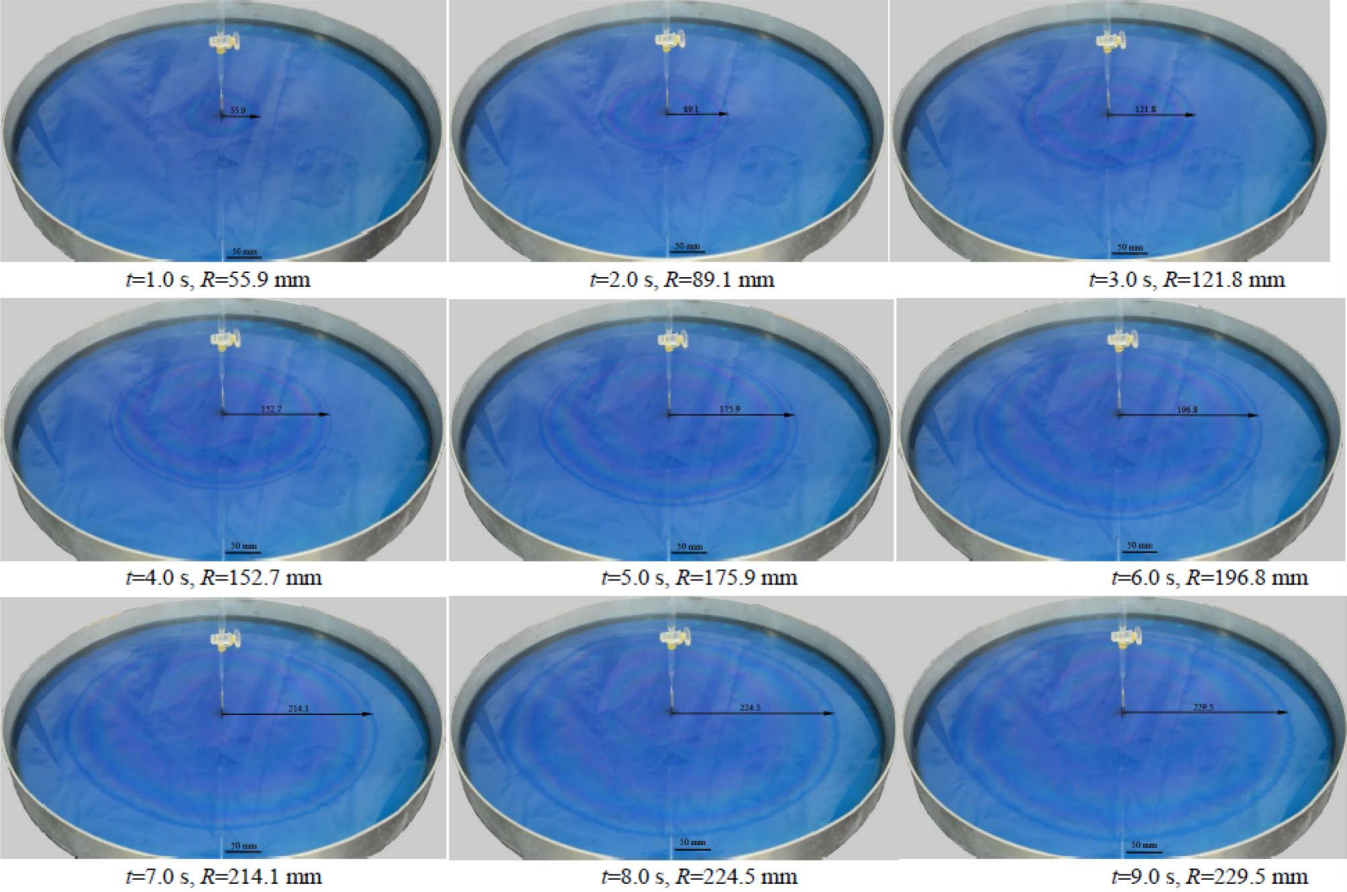
The actual spreading scenes of aqueous film formed by C4-Br/CH_3_(CH_2_)_5_NaO_3_S solution spread on the surface of cyclohexane at different times at 25 °C.

The actual spreading situations of the C4-Br/CH_3_(CH_2_)_5_NaO_3_S solution on the surfaces of cyclohexane, *n*-heptane, aviation kerosene, solvent naphtha and diesel oil 0# were investigated in this work, respectively. The results showed that the aqueous film expanded outwards in a circle with the guiding device as the center. This was the first time to directly observe the two-dimensional spread of water film on the oil surface using a high-definition camera. The spreading scenes of C4-Br/cyclohexane system at different times at 25 °C were shown in Fig. 4.

#### Evaluation of the experimental results

The model for surface-tension-viscous regime could well predict the unidimensional spreading process of the aqueous film formed by surfactant solution spread on the hydrocarbon oils according to our previous study^28^. For two-dimensional spread, the spreading process could be described by the following equation^14^.

1 $$R = k\left( {\frac{{S^{2} }}{{\rho^{2} \upsilon }}} \right)^{0.25} t^{0.75} = k\beta t^{0.75} = At^{0.75}$$

where *R* was the radius of spread, cm; *k* was a constant characteristic of the given system; $S = \gamma_{o} - \left( {\gamma_{w} + \gamma_{o/w} } \right)$, was the spreading coefficient, where *γ*_*o*_ and *γ*_*w*_ were the surface tension of the hydrocarbon oil and the aqueous solution respectively, *γ*_*o/w*_ was the interfacial tension of the aqueous solution against hydrocarbon oil, mN/m; *υ* was the kinematic viscosity of fuel, cm^2^/s; *ρ* was the density of fuel, g/cm^3^; *t* was the spreading time, s; $\beta = \left( {\frac{{S^{2} }}{{\rho^{2} \upsilon }}} \right)^{0.25}$, was a self defined parameter related to the nature of the research system, cm/s^0.75^; *A* = *kβ*, cm/s^0.75^. The pre-exponential factor *A* could be determined by nonlinear fitting using Origin to the experimental data. Since the *β* values had been measured in our previous work^28^, the *k* values in the C4-Br/oil systems could be obtained.

The *A*, *β* and *k* values in the different C4-Br/oil systems were listed in Table 1. It can be seen from Table 1 that *k* value of each system is different. The minimum value is 1.51 in the C4-Br/diesel oil 0# system and the maximum one is 1.82 in the C4-Br/solvent naphtha system. Two-dimensional surface tension spread. According to the study of Fay and Hoult^14^, the *k* value of two-dimensional surface tension spread is 2.3, but the data are not verified by the experiments. Camp^36^ has compiled many theoretical and experimental values of *k* in different spreading systems and suggests that *k* can range in magnitude from 0.665 to 1.52 theoretically. However, the experimental values obtained from the spreading of single or multi-component oils and alcohols have ranged between 1.1 and 2.1. The results in this work are generally consistent with the researches of the pioneers.

**Table 1:** The values of *A*, *β* and *k* in the different spreading systems at 25 °C.

System	C4-Br/cyclohexane	C4-Br/*n-*heptane	C4-Br/aviation kerosene	C4-Br/solvent naphtha	C4-Br/diesel oil 0#
*A* (cm/s^0.75^)	5.17	4.51	4.69	4.61	3.67
*β* (cm/s^0.75^)	3.06	2.50	2.94	2.53	2.43
*k*	1.69	1.80	1.60	1.82	1.51

**Figure 5:**
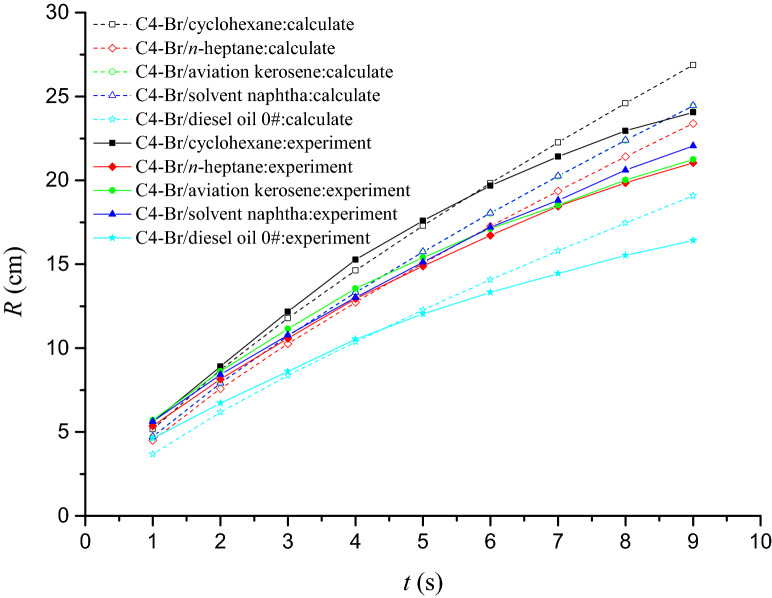
The *R*-*t* graphs of C4-Br/oil systems obtained from experiment and theoretical calculate.

The two-dimensional spreading radius and time of the C4-Br/oil systems obtained from experiment and calculated from Eq. (1) were both plotted in Fig. 5. According to Fig. 5, we can found that the spreading radius for all of the C4-Br/oil systems increase with the extension of spreading time. At the beginning, the actual spreading radius is a little larger than the theoretical value. However, as the spreading goes on, the theoretical value gradually exceeds the experimental one. The longer the spreading time is, the larger the gap between theoretical and experimental value is. This is consistent with the results of one-dimensional spreading study^28^. The cause of this phenomenon is that, as a driving force, the gravity together with the spreading coefficient accelerates the spread at the beginning. As the spreading progresses, the reason for the disagreement between theory and experiment becomes that the spreading coefficient is not constant, but depends on time. The concentration of the surfactant in the aqueous film at expanding front is lower than that in the bulk due to the reestablishment of the equilibrium by diffusion associated with demicellization in the bulk is not fast enough as compared with the time scale of expansion^37^. This leads to a higher surface and interracial tensions, implying that the spreading coefficients in the actual spreading process are reduced. The larger the spreading radius is, the farther the spreading front is from the bulk, and the more obvious the reduction of spreading coefficient is.

The quality of the correlation using Eq. (1) for the investigated system was measured by the root-mean-square deviation (*RMSD*) and the *RMSD* value was 1.55 for C4-Br/cyclohexane system, 1.07 for C4-Br/*n*-heptane system, 1.54 for C4-Br/aviation kerosene system, 1.21 for C4-Br/solvent naphtha system and 1.64 for C4-Br/diesel oil 0# system, respectively. The smaller *RMSD* indicate a good agreement between the experimental and theoretical values.

### Sealing performance of surfactant solution film

#### Determination of aqueous film thickness

The thickness of aqueous film was one of the key factors affecting its sealing performance. In fact, the film thickness was not a fixed value, but a range determined by the minimum and the maximum thickness respectively for a given surfactant/oil system. It was necessary to determine the thickness of aqueous film in order to study the suppress effect of film on the oil evaporation. The film thickness ranges for the C4-Br/cyclohexane, C4-Br/*n*-heptane, C4-Br/aviation kerosene, C4-Br/solvent naphtha and C4-Br/diesel oil 0# systems were listed in Table 2.

**Table 2:** The thickness range of aqueous film in the different spreading systems at 25 °C.

System	C4-Br/cyclohexane	C4-Br/*n-*heptane	C4-Br/aviation kerosene	C4-Br/solvent naphtha	C4-Br/diesel oil 0#
*V*_min_ (µL)	33.5	31.2	82.2	27.2	63.8
*V*_max_ (µL)	561.2	477.5	607.4	480.5	612.2
*σ*_min_ (µm)	1.3	1.2	3.2	1.1	2.5
*σ*_max_ (µm)	22.1	18.8	23.9	18.9	24.1

As shown in Table 2, the film thickness range of each spreading system is different. The *σ*_min_ ranges from 1.1 to 3.2 µm and the ranges from 18.8 to 24.1 µm. Tuve^38^ pointed out that the thickness of the film formed by AFFF is about 10–30 µm. This may be due to that AFFF has more chemical components and higher viscosity, so the aqueous film formed by AFFF is a little thicker. In order to compare the sealing properties of the surfactant solution for different fuels, the film thickness was determined uniformly as 10.0 μm in this work.

#### Relationship between sealing performance and time

**Figure 6:**
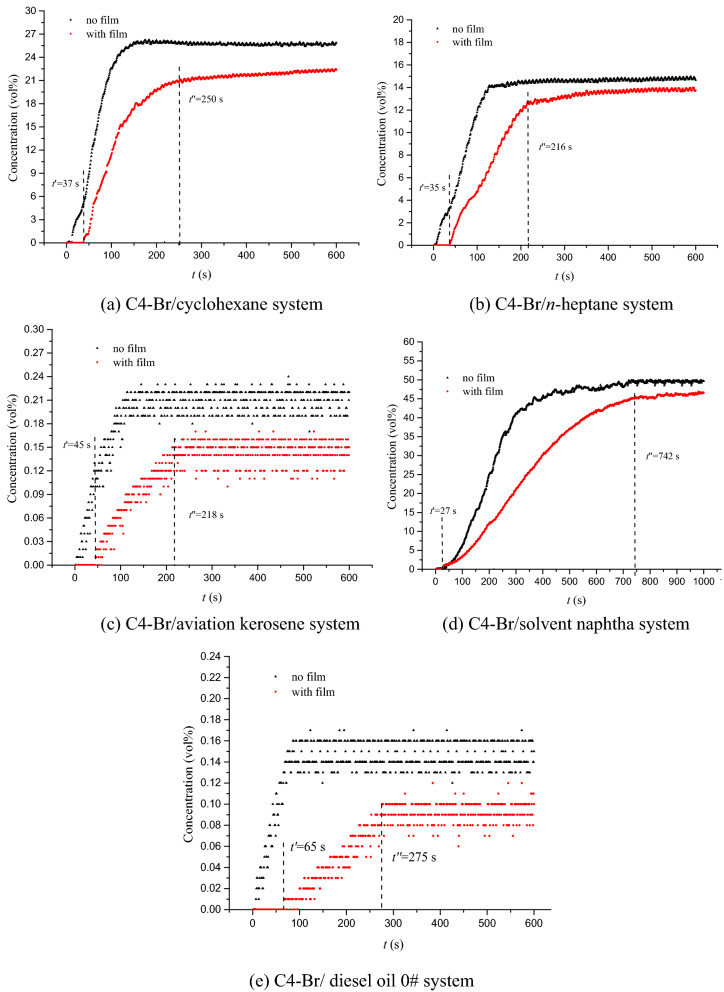
The volume concentration of the fuel vs. time with and without sealing by aqueous film at 25 °C: *t’* was the absolute sealing time and *t*” was the loss sealing time.

The aqueous film form by AFFF could act as a vapor barrier to deplete the supply of fuel to the flame and prevent reignition of the fire once it had been extinguished in fire-fighting applications. The retardation of aqueous film on fuel evaporation was studied in this work by measuring the volume concentration of hydrocarbons above the fuel at 25 °C. The vapor volume concentrations for cyclohexane, *n*-heptane, aviation kerosene and diesel oil 0# ranged from 0 to 600 s and 1000 s for solvent naphtha with and without aqueous film were investigated, respectively. The experimental results were shown in Fig. 6, where *t’* was the absolute sealing time when the oil vapor had just been detected and *t*” was the loss sealing time when the vapor concentration did not increase obviously.

From Fig. 6, we can found that the oil vapor concentration of each system without film increased first and then remained unchanged after reaching saturation. However, for the systems with film, the oil vapor concentration remained at 0 for a period of time before increasing, indicating a good sealing performance of the aqueous film formed by surfactant solution. The blank period of oil vapor concentration ranges from 27 to 65 s for different system, and the stronger the volatility, the shorter the sealing time. With the gradual destruction of the film, the oil vapor concentration increases and finally reaches saturation. Specifically, as shown in Fig. 6c,e, due to the high flash points of aviation kerosene (about 38 °C) and diesel oil 0# (about 56 °C), the oil vapor concentrations at 25 °C are very small (less than 0.24%) for the C4-Br/aviation kerosene and C4-Br/ diesel oil 0# systems. Additionally, limited by the test accuracy, the data has a certain degree of vibration. However, the trends of oil vapor concentrations can be seen still clearly. It is worth noting that the concentration of oil vapor with film is always lower than that without film even when the evaporation is saturated. This is due to the formation of micelles with lipophilic group outward when the surfactant solution is added into the hydrocarbon fuel. The micelles dissolved in the oil phase can not only reduce the effective concentration of hydrocarbons slightly, but also bind the liquid molecules.

## Conclusion

A method for studying the two-dimensional spreading properties and sealing characteristics of surfactant solution on oil surface was provided in this work. The actual situation of the aqueous film formed by fluorosurfactant solution spreading on oil surface in axisymmetric geometry was observed directly using HD camera for the first time. The spreading radius for all of the C4-Br/oil systems increase with the extension of spreading time and the results obeyed the model of surface-tension- viscous regime. The results of sealing experiments showed that the aqueous film could absolutely seal the oil surface for 27–65 s and the sealing effect would be lost after 216–742 s for different systems. The stronger the volatility was, the shorter the sealing time was. Additionally, the volume percentage of oil vapor with film was always lower than that without film even when the evaporation was saturated.

It should be noted that there are many factors can affect the spreading and sealing performance of aqueous film formed by surfactant solution on the oil surface. The drop acceleration and concentration of surfactant solution are the two important factors not involved in this work. In addition, AFFF is spread on the oil surface with a high temperature and disturbance in the actual fire extinguishing process. Therefore, it is very important to study the influence of temperature and turbulence on the spreading kinetics of surfactant solution on the oil surface.
